# Identification of novel drugs to target dormant micrometastases

**DOI:** 10.1186/s12885-015-1409-4

**Published:** 2015-05-14

**Authors:** Robert E. Hurst, Paul J. Hauser, Youngjae You, Lora C. Bailey-Downs, Anja Bastian, Stephen M. Matthews, Jessica Thorpe, Christine Earle, Lilly Y. W. Bourguignon, Michael A. Ihnat

**Affiliations:** 1 Departments of Urology, Oklahoma University Health Sciences Center, 940 S. L. Young Blvd, Oklahoma City, OK 73104 USA; 2 Biochemistry & Molecular Biology, College of Medicine, Oklahoma University Health Sciences Center, 940 S. L. Young Blvd, Oklahoma City, OK 73104 USA; 3 Department of Pharmaceutical Sciences, College of Pharmacy, Oklahoma University Health Sciences Center, 940 S. L. Young Blvd, Oklahoma City, OK 73104 USA; 4 Stephenson Cancer Center, Oklahoma University Health Sciences Center, Oklahoma City, OK 73104 USA; 5 DormaTarg, Inc., 940 S.L. Young Blvd, Suite 118, Oklahoma City, OK 73104 USA; 6 Department of Medicine, University of California, San Francisco and the VA Medical Center, 4150 Clement St., San Francisco, CA 94121 USA

**Keywords:** Micrometastases, Dormancy, Targeted therapy, Metastasis prevention

## Abstract

**Background:**

Cancer-specific survival has changed remarkably little over the past half century, mainly because metastases that are occult at diagnosis and generally resistant to chemotherapy subsequently develop months, years or even decades following definitive therapy. Targeting the dormant micrometastases responsible for these delayed or occult metastases would represent a major new tool in cancer patient management. Our hypothesis is that these metastases develop from micrometastatic cells that are suppressed by normal extracellular matrix (ECM).

**Methods:**

A new screening method was developed that compared the effect of drugs on the proliferation of cells grown on a normal ECM gel (small intestine submucosa, SISgel) to cells grown on plastic cell culture plates. The desired endpoint was that cells on SISgel were more sensitive than the same cells grown as monolayers. Known cancer chemotherapeutic agents show the opposite pattern.

**Results:**

Screening 13,000 compounds identified two leads with low toxicity in mice and EC_50_ values in the range of 3–30 μM, depending on the cell line, and another two leads that were too toxic to mice to be useful. In a novel flank xenograft method of suppressed/dormant cells co-injected with SISgel into the flank, the lead compounds significantly eliminated the suppressed cells, whereas conventional chemotherapeutics were ineffective. Using a 4T1 triple negative breast cancer model, modified for physiological metastatic progression, as predicted, both lead compounds reduced the number of large micrometastases/macrometastases in the lung. One of the compounds also targeted cancer stem cells (CSC) isolated from the parental line. The CSC also retained their stemness on SISgel. Mechanistic studies showed a mild, late apoptotic response and depending on the compound, a mild arrest either at S or G_2_/M in the cell cycle.

**Conclusions:**

In summary we describe a novel, first in class set of compounds that target micrometastatic cells and prevent their reactivation to form recurrent tumors/macrometastases.

**Electronic supplementary material:**

The online version of this article (doi:10.1186/s12885-015-1409-4) contains supplementary material, which is available to authorized users.

## Background

In spite of billions spent on research, cancer–specific survival is remarkably unchanged for many cancers over the past 50 years, even with the newer “targeted” therapies [1, 2]. This is in part due to the main models of drug development and research being based on primary tumors [3, 4], whereas the fatal event in patients is development of therapy–resistant metastatic tumors [5]. Metastasis, which is often undetected at the time of diagnosis, is responsible for the death or 90 % patients who succumb to their cancer [6]. Moreover, increasing evidence demonstrates that metastasis can be an early event [7, 8], which suggests that early detection of primary tumors may not be the panacea that some have hoped, at least in some tumors. Further, given that surgery or radiation to a primary tumor can actually enhance the growth of secondary tumors, these conventional treatments could actually end up shortening lives, not increasing them [9, 10].

One of the main barriers that has inhibited drug development targeting metastasis has been a general unavailability of good models and, in particular, of an *in vitro* screening system capable of identifying candidate compounds. We present here a new *in vitro* model for identifying compounds that target metastasis at its most vulnerable and rate-limiting step, which is the escape of micrometastatic cells from the suppressive effects of the normal extracellular matrix [11]. Every metastatic tumor starts as a single micrometastatic cell or small avascular group of cells. These can often be seen in various tissues of cancer patients [12]. Interestingly, these cells may not begin to grow immediately. If they fail to die by apoptosis they can remain in a quiescent or suppressed state for months or years before eventually escaping to form metastatic tumors [13–19]. Our hypothesis is that these cells are suppressed by the presence of normal extracellular matrix (ECM), which has been shown to function as a “gatekeeper” for tumorigenesis [11, 20]. The awakening and growth of micrometastatic cells therefore is the committed step in metastasis, and if such suppressed cells could be targeted effectively, a breakthrough in cancer therapy could result. ECM-suppressed cancer cells may also be a factor in local recurrence [21–26]. The ECM-suppressed cell is also an attractive therapeutic target because they are single cells and do not display the heterogeneity seen in the primary tumor and macrometastases [27]. Suppressed or dormant cancer cells appear to be resistant to conventional chemotherapeutic agents (regardless of whether an eventual metastatic tumor arising from them is drug-sensitive) as we and others have shown experimentally and because chemotherapy does not generally prevent delayed metastasis [28–30]. Therefore, while new drugs may attack primary tumors and even cause dramatic shrinkage, most cancer patients who die of their disease have metastatic progression [6, 31].

Several years ago, we observed that cancer cells grown on a gel–forming product derived by pepsin digestion of porcine small intestine submucosa (SISgel), exhibited a suppressed, normalized phenotype involving loss of key malignant properties such as invasiveness [32]. Lower grade bladder cancer cells grown on SISgel even formed a layered structure reminiscent of normal epithelium [32]. We also showed that cancer cells grown on either Matrigel, where they fully expressed their malignant phenotype, or SISgel, where they were suppressed, were several–fold more resistant to conventional cancer therapeutics than were the corresponding cells grown on a plastic surface in conventional tissue culture, which is the basis for most initial drug discovery. In other words, cancer cells grown on any matrix were more resistant to known cancer therapeutics than they were in conventional tissue culture [30]. We reasoned that a drug that specifically targeted cancer cells suppressed by normal ECM would show the opposite pattern, that is they would be more resistant when grown on a plastic surface and more sensitive when grown on normal ECM. With this readout in mind, we developed a 96-well format screen in which the cells were grown on SISgel in one plate and on a plastic surface as actively growing monolayers in a second plate. A “hit” was defined as cancer cells being more sensitive to the test compound when grown on the suppressive SISgel than on the plastic surface. We herein describe the results of screening two chemical libraries and testing the hits in an *in vivo* mouse model of a suppressed tumor in flank xenografts as well as in natural metastasis in a syngeneic mouse model that does not involve SISgel. The results demonstrate that cancer cells on normal extracellular matrix can, indeed, be targeted and that this targeting could result in new treatments to prevent metastases from developing from micrometastatic cells.

## Methods

### Screening of compound libraries

The libraries were obtained from the NCI (Diversity Library I) comprised of 2,918 compounds and a 10,000 compound diversity library from ChemBridge, Inc. (DiverSet-EXP). The NCI library is no longer available in the form used here, but how it was constructed is described (http://dtp.nci.nih.gov/branches/dscb/diversity_explanation.html). The ChemBridge library is a subset (Set Code NM1024) of the full 50,000 compound library (http://www.chembridge.com/screening_libraries/diversity_libraries/). Compounds were diluted to 100 μM before use. SISgel was prepared as described previously [33, 34] from powdered porcine small intestine submucosa obtained from Cook Biotech (W. Lafayette, IN). It is a natural product that is used extensively in tissue engineering as a bioscaffold [34]. J82 bladder cancer cells and other cell lines were obtained from the ATCC (Bethesda, MD). The basic principle is that cells grown on any ECM are, in general, expected to show higher resistance to anticancer compounds than when grown on plastic [30, 35–37]. Therefore, compounds that show a higher activity toward cancer cells on a normal ECM than on a plastic surface would be candidates to target suppressed, micrometastatic cells. Efficacy *in vitro* was assessed by plating 30,000 cells in 50 μL of high glucose DMEM with penicillin-streptomycin and 10 % fetal calf serum on SISgel in a 96-well plate containing 50 μL of gelled SISgel per well. Cells were allowed to attach and assume the SISgel phenotype for 48 h. A matching plate without SISgel was prepared 24 h later by pipetting 3,000 cells in 100 μL of high glucose DMEM with penicillin-streptomycin and 10 % fetal calf serum into each well of 96 well plate. The cells were allowed to attach and grow for 24 h. These numbers of cells gave approximately equal replication rates at the time drug was added for cells grown on the plastic surface or SISgel as determined by the fraction of S-phase cells identified by flow cytometry [30]. The cells were exposed to drug in fresh medium for 48 h, at which time the cell number was determined using the CFDA-AM cellular esterase proliferation assay (cat. #C1354, Life Technologies). For screening the results are reported as the ratio of cell count of cells on the plastic surface to the cell count on SISgel. An initial difference of 1.5 fold identified potential hits. All potential hits were confirmed in triplicate. Those compounds that replicated were confirmed by a full dose–response study yielding an EC_50_ using five wells per drug concentration. Data were analyzed using nonlinear regression to a sigmoid curve with Prism 4 (GraphPad Software, Inc., LaJolla, CA). Potential hits that were confirmed by dose–response using J82 were also tested with MDA-MB-435 breast cancer, PC-3 metastatic prostate cancer, Capan1 pancreatic cancer and the J82 bladder cancer cell lines.

### Spheroid formation and self-renewal assays

Sphere formation to further increase the fraction of cancer stem cells was induced by suspending CD44v3highALDH1high 4T1 cells in 1:1 Matrigel/basal medium in a total of volume of 100 μl. Samples (5 × 10^4^ cells) were then plated around the rims of wells in a 12-well plate and allowed to solidify at 37 °C for 10 min before 1 ml basal medium (with B27 plus 20 ng/mL EGF, 10 ng/mL FGF and 4 μg/mL insulin) was added. Medium was replenished every 3-days. Ten days after plating, spheres (tight, spherical, nonadherent masses >40 μm in diameter) per well were counted, and at least 100 spheres per group were measured. The number of spheres containing CD44v3highALDH1high 4T1 cells in each well and expressed as sphere forming units (SFU) as described previously [38]. The number of SFUs per well was counted in triplicate wells for each condition.

To recover CD44v3highALDH1high 4T1 cells from the spheres, Matrigel–containing wells were treated with 1 mg/ml Dispase solution (Gibco). Spheres were then digested with trypsin and 0.05 % EDTA. CD44v3highALDH1high cells dissociated from spheres were counted by hemacytometer and replated to generate spheres of next generation. Serial passage of individual spheres was regularly performed in order to verify self-renewal capability of cells associated the spheres. Measurement of growth for CD44v3highALDH1high cells (dissociated from spheres) was also performed by incubating these cells in serum-free RPMI-1640 medium for 3-weeks using MTT-based growth assay as described previously [38].

### Tumor cell growth inhibition assays

To analyze tumor cell growth properties, sphere-derived CD44v3highALDH1high 4T1 cells were incubated in basal medium (with B27 plus 20 ng/mL EGF, 10 ng/mL FGF and 4 μg/mL insulin). Medium was replenished every 3-days. Twenty-one days after plating, the number of cell growth was then counted under a microscope at low magnification. In some cases, these sphere-derived CD44v3highALDH1high 4T1 cells were also treated with DT compounds for 5 days or doxorubicin (range 1 μM to 1 mM) for 5 days at 37 °C. The CellTiter-Glo® Luminescent Cell Viability Assay (Promega, Madison, WI) was utilized to determine the number of metabolically active cells based on the quantification of adenosine triphosphate (ATP). The percentage of absorbance relative to untreated controls was plotted as a linear function of drug concentration. The 50 % inhibitory concentration (IC_50_) was identified as the concentration of drug required to achieve a 50 % growth inhibition relative to untreated controls.

### Flank xenograft model

The efficacy of DT320 was assessed using a flank xenograft model of ECM-suppressed cancer cells that we developed [33], as well as an orthotopic model of “triple-negative” breast cancer [39]. All animal protocols were reviewed and approved by the OUHSC Institutional Animal Care and Use Committee according to criteria established by the NIH Office of Laboratory Animal Welfare, and animals were housed 5 per cage with enrichment (tubes) and wood chips in the AALAC-accredited OUHSC Animal Facility with a 12 h light/dark cycle. Animals had access to water and food *ad libatum.* Animals were monitored daily by trained facility personnel. In the flank model, GFP-labeled cells were co-injected with SISgel, which produces a suppressed, normalized phenotype that persists after the SISgel is resorbed, but which can re-emerge into active tumor growth after 30–60 days [33]. Animals were all female *nu/nu* mice (NCI National Laboratory Frederick/Charles River animal production program). An optimized number of 5 × 10^5^ GFP-labeled MDA-MB-435 cells in 100 μL DMEM medium, without additives, were mixed with 100 μL of SISgel and were kept on ice until injection to prevent polymerization of the SISgel. The time the cells were kept in the cold was minimized. The cell-SISgel mixture was mixed by drawing it into a 1 mL tuberculin syringe with a 23 ga. needle. The mixture of 200 μL was injected into the flanks, two per mouse, just anterior to the rear legs on either side of the spine. The MDA-MB-435 flank model animals were treated with 45 mg/kg of DT-310 and DT-320 three times weekly, while gemcitabine was administered intraperitoneally twice weekly at 75 mg/kg starting 5 days after tumor implantation. Treatment was initiated at the MTD of 75 mg/kg, three times weekly, intraperitoneally, seven days after injection. The cells injected into the flanks remained as a non-growing spot, as assessed by fluorometry of the spots and the lack of mitoses seen in tissue sections, for about 3–5 weeks before some began resuming malignant growth [33]. We are aware of the controversy concerning the identity of MDA-MB-435 cells [40], but these were obtained by one of us (MAI) from the laboratory of Janet Price in the mid1990s before any contamination. In addition, genetic analysis demonstrated these were clearly breast cancer cells.

### Orthotopic 4T1 model

Efficacy was also tested in an orthotopic, syngeneic mouse model that exhibits natural metastasis following a reproducible dormant period for micrometastatic cells in the lung and other tissues (39). 4T1 Luc2-GFP cells (PerkinElmer, Waltham, MA) were cultured in DMEM high glucose media with 2 mM pyruvate, 2 mM glutamine, 2 % Pen/Strep and 10 % Cosmic Calf Serum (Mediatech, Manassas, VA). To prepare cells for injection they were removed from flasks, counted using the TC10 counting system (BioRad, Hercules, CA) and checked for viability. Cells were then washed with PBS, pelleted and taken up at a concentration of 75,000 cells/ml; 100 μl of cell suspension (7,500 cells) was injected subcutaneously (bevel side up) into mammary fat pad #4 of 8 weeks old female BALB/c mice (NCI National Laboratory Frederick/Charles River animal production program). All animals were weighed and tumors were measured using calipers three times weekly. Beginning one week after implantation, which is when micrometastatic cells begin to arrive in the lungs [39], animals were treated three times per week with intraperitoneal DT310 or DT320 at their NOAEL dose of 75 mg/kg three times week dissolved in saline or by osmotic pump. The osmotic pumps (Alzet #2004) delivered an NOAEL dose of 23.4 and 26.7 μg/h of DT310 and DT320, respectively, for 4 weeks and were implanted as directed subcutaneously posterior to scapulae. Docetaxel was delivered once weekly intraperitoneally at its MTD of 15 mg/kg and doxorubicin delivered once weekly at its MTD of 1 mg/kg in 5 % *N*-Methyl-2-pyrrolidone, 5 % Solutol HS, and 90 % saline. GFP positive cells were imaged with excitation of GFP emission using a Leica Model Z16 APO fluorescence microscope equipped for a wide field, a large depth of field and 0.57–9.2X zoom capability. GFP-positive objects were counted and scored as to whether they were large micrometastases, clumps of less than ten cells without a visible vasculature, as determined by co-injection of tetramethylrhodamine labeled dextran (2 million MW).

### Identification of analogs and potential targets

(InhibOx, Oxford, UK) In brief, for each query compound the “2D” chemical structures were used to create a description of the compounds in a computational format called SMILES, which is a simple line notation. For each of these SMILES, 3D conformational models were generated, so that each compound is represented as a set of low-energy conformations. From these, ElectroShape descriptors were created that enable the fast database searching. Standard partial charge parameters for the electrostatic component were used as described [41–43]. Potential targets also were identified from known targets of the compounds identified as active analogs from the algorithmic approach.

#### RPPA analysis

MDA-MB-435 and T24 cells were treated with DT320 or DT321, a less active analog, for 4, 8, 18 and 48 h at their EC_50_. Cell lysates were collected for reverse phase protein array (RPPA) analysis and expression compared to the same cells treated with doxorubicin, cisplatin, docetaxel and pemetrexed at their EC_50_ concentrations. DT321, a close structural analog of DT320, was used to confirm the results of DT320.

### Western blot analysis

Protein content of selected proteins identified by reversed phase protein array (see supplementary materials) as being involved mechanistically in the response to DT320 was assayed by Western blot as described [44] using the following antibodies. The following antibodies were purchased from Cell Signaling Technology (Beverly, MA) and diluted per manufacturer’s directions: Rabbit mAb Anti-Phospho-AKT (Ser473) (D9E) XP® #4060, Rabbit mAb anti-p38 MAPK (D13E1) XP® #8690, Mouse mAb anti-Phospho-SAPK/JNK (Thr183/Tyr185) (G9) #9255, Rabbit mAb anti-Phospho-CHK1 (Ser345) (133D3) #2348, Rabbit polyclonal anti-Phospho-CHK2 (Thr68) Antibody #2661, Rabbit polyclonal anti-BAX Antibody #2772, Rabbit polyclonal anti-Vinculin Antibody #4650, Mouse mAb anti-Cyclin D1 (DCS6) #2926, Rabbit mAb anti-Cyclin E2 (D52F9) b #3741, Mouse mAb anti-Cyclin A2 (BF683) #4656, Mouse mAb anti-Cyclin B1 (V152) #4135, Rabbit mAb anti-cleaved PARP (Asp214) (D64E10) XP® #5625, Rabbit mAb anti-cleaved Caspase-3 (Asp175) (5A1E) #9654, Rabbit mAb anti-Nanog (D73G4), #4903; Rabbit mAb anti-Oct4 (C52G3) #2890; and Rabbit mAb anti-Sox2 (D6D9) #3579. Specificity of each antibody is illustrated on the company’s web page. For negative controls, pre-immune rabbit IgG was used. No signal was detected in the control IgG samples. No intensities or contrast were modified, but the appropriate bands were cut out using PhotoShop to prepare composite gel images.

#### Cell cycle analysis

MDA-MB-435 cells were treated with DT310 and DT320 at 100 μM for 24 h. Cells were run in triplicate with 1 μM of docetaxel being used as a positive control. Cells were rinsed twice in PBS, then trypsinized, collected and centrifuged. They were resuspended in 1:1 PBS:100 % EtOH and centrifuged at 1200 RPM for 5 min. Cells were stained with 25 μg/ml propidium iodide solution for 60 min at 37 °C, and resuspended in 600 μL of PBS for analysis. Cells were analyzed with a FACSCalibur (BD) flow cytometer, and the cell cycle profile was determined with ModFit v.2 (Verity Software, Topsham, ME) cell cycle analysis software.

#### Effect of DT compounds on cancer stem cells

##### Sorting tumor–derived 4T1 cell populations by multicolor fluorescence–activated cell sorter (FACS)

A stem cell–enriched cell population was prepared as described previously [38] by sorting for ALDH1 and CD44v3. The identification of aldehyde dehydrogenase1 (ALDH1) activity from tumor–derived 4T1 Luc2-GFP cells (PerkinElmer, Waltham, MA) was conducted using the ALDEFLUOR kit (StemCell Technologies, Durham, NC). Specifically, tumor cells were suspended in ALDEFLUOR assay buffer containing ALDH1 substrate (BAAA, 1 mol/L per 1 × 10^6^ cells) and incubated for 30 min at 37 °C. As a negative control, 4T1 cells were treated with a specific ALDH1 inhibitor, 50 mM diethylaminobenzaldehyde (DEAB). Next, for labeling cell surface marker, tumor–derived 4T1 cells were suspended in 100 μl ALDEFLUOR buffer followed by incubating with 20 μl allophycocyanin (APC)-labeled anti-CD44v3 antibody (recognizing the v3-specific domain of CD44) or APC-labeled normal mouse IgG (as a control) (BD Bioscience, San Jose, CA) for 15 min at 4 °C. For FACS sorting, tumor cells were incubated in PBS buffer followed by FACS (BD FACS Aria llu, BD Bioscience, San Jose, CA) sorting using dual-wavelength analysis as described previously [35]. The parental cell line contained 2 % CD44v3highALDH1high cells but the final sorted cells contained 18 % CD44v3highALDH1high cells, a 9-fold enrichment.

## Results

### Identification of lead compounds

In the screen of approximately 3,000 compounds in the “diversity set” from the National Cancer Institute (NCI), a total of seven potential hits were identified. Of these seven, only two compounds showed activity in multiple cell lines and a distinct difference in the EC_50_ between cells grown on the plastic surface or SISgel as determined from the full dose–response data. These two compounds were named DT310 and DT320 and are described in Table 1, along with two compounds identified in a second library screen of 10,000 diverse drug–like compounds. The EC_50_ values shown in Table 1 indicate that DT310 and DT320 compounds are more effective than doxorubicin on breast cancer cells grown on SISgel and are thus selective for cancer cells in a suppressed state. This selectivity is not simply an effect of growing the cells on a gel surface instead of a plastic surface, because the same effect is not seen with Matrigel, and the drugs show similar selectivity against cells on SISgel as opposed to either Matrigel a plastic surface is observed. Rather DT compounds have true selectivity for normal ECM-suppressed cancer cells (Table 2). Table 3 shows that the finding seems to be generally true for several cancer cell lines. The results are not specific to a given cell type or cancer of origin, when compared to conventional chemotherapeutic agents (Table 3). DT310 and DT320 show a higher efficacy than did the conventional agents in the *in vitro* model. Several conventional chemotherapeutics were used for comparison to demonstrate the unique mechanism of action of the DT agents. The two screens yielded different kinds of compounds. The compounds identified from the NCI diversity set (DT310 and 320) had low toxicities to mice with toxicity in the form of weight loss only appearing at doses of 65 to 75 mg/kg. In contrast, the two compounds identified from the Chembridge diversity library (DT340 and 350) were both highly toxic to mice at doses of less than 10 mg/kg. The toxic compounds were not considered further for development.

**Table 1:** Relative potency of hits against MDA-MB-435 cells on SISgel (S) and plastic (P) and MTD vs doxorubicin

Designation	Chemical name, CAS number and link to structure	MW	EC_50_-P (μM)	EC_50_-S (μM)	SI^a^	MTD (mg/kg)
DT310	4-(1-naphthalenylhydrazinylidene) -3-oxonaphthalene-2,7-disulfonic acid 5858-33-3. http://www.chemnet.com/cas/es/5858-33-3/Bordeaux%20R.html	502	86.5	35.9	2.4	65
DT320	4,5-Dihydroxy-3-(1-naphthalenylazo)-2,7-naphthalenedisulfonic acid disodium salt 5850-63-5 http://www.chemnet.com/cas/en/5850-63-5/Pontacyl-violet.html	518	78.0	8.7	9.0	75
DT340	10-(2,3,4-trimethoxyphenyl)-6,7,8,10-tetrahydro-5H-indeno[1,2-b]quinoline-9,11-dione 669753-40-6 http://www.hit2lead.com/result.asp?search=87085397	417	219	117	1.9	<10
DT350	2,2,4-trimethyl-1,2-dihydro-6-quinolinyl (4-methoxyphenyl)-acetate 376621-78-2 http://www.hit2lead.com/result.asp?search=43317184	337	1831	270	6.8	<10
Doxorubicin	7S,9S)-7-[(2R,4S,5S,6S)-4-amino-5-hydroxy-6-methyloxan-2-yl]oxy-6,9,11-trihydroxy-9-(2-hydroxyacetyl)-4-methoxy-8,10-dihydro-7H-tetracene-5,12-dione 23214-92-8	544	47.8	45.4	1.05	1

^a^Ratio of EC_50_ of cells grown on a plastic surface (P) versus cells grown on SISgel (S) (EC_50_-P/ EC_50_-S) in the presence of drug

**Table 2:** EC_50_ (μM) values of DT320 for different cancer cell lines grown on Matrigel (fully malignant phenotype) vs SISgel (suppressed phenotype)

Cell line	Matrigel	SISgel	Ratio	*p*
MDA-MB-435 (breast)	48.2	20.0	2.4	<0.01
U251 (glioblastoma)	95.0	64.6	1.5	N.S.
DU145 (prostate)	183.2	45.7	4.0	<0.001
AGS (gastric)	104.4	45.1	2.3	<0.01

**Table 3:** Comparison of EC_50_ values (μM) of DT320 vs conventional agents on different cancer cell lines

Cell Line	DT320	DT320	Conventional agent	Conventional agent
	SISgel	Monolayer	SISgel	Monolayer
MDA-MB-231 (breast)	8.7 ± 1.1	78.1 ± 7.1	47.8 ± 4.0 (D)	45.4 ± 1.9 (D)
PC-3 (prostate)	19.7 ± 2.0	41.5 ± 4.1	102.2 ± 7.0 (D)	104.1 ± 6.1 (D)
J82 (bladder)	30.9 ± 4.8	71.2 ± 8.1	>300 (C)	102.3 ± 10.2 (C)
Capan-1 (pancreatic)	22.9 ± 4.8	80.1 ± 10.2	83.1 ± 10.7 (G)	78.2 ± 9.8 (G)

Data represent n = 6-8 from three separate experiments. D = doxorubicin, G = gemcitabine, C = cisplatin

### Efficacy *in vivo*

**Fig. 1:**
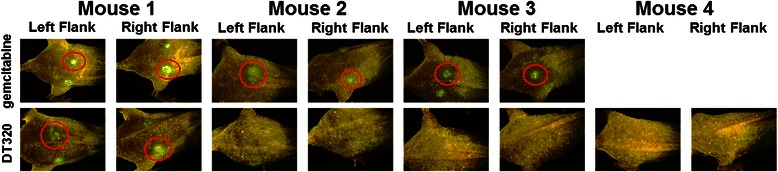
DT320 induces complete regression of suppressed MDA-MB-235 GFP tumors. For the *in vivo* efficacy of DT320 in 500,000 MDA-MB-435-GFP cells were implanted together with SISgel in the flank of NCr nu/nu athymic mice. One week after inoculation, DT320 was administered i.p. at 45 mg/kg three times weekly for 3 weeks. In 6 of 8 flanks treated with DT320, suppressed tumor cell spots disappeared, while the tumor cell spots in the 6 flanks treated with gemcitabine (75 mg/kg, twice weekly) remained present. (For space considerations, the other two xenografts not responding to gemcitabine were not shown). Images are taken at 4 weeks after implantation. The difference in response was statistically significant at p = 0.0097 using Fisher’s Exact Test

The flank xenograft model used in these studies provides a model of “dormancy” in which suppression is induced by normal ECM. The difference between the *in vitro* and *in vivo* models is that the SISgel is resorbed by the mouse, leaving a small green-glowing spot that likely consists of ECM-suppressed cells that can eventually break out of dormancy and begin growing as an aggressive tumor (Fig. 1). The animals that were treated with 75 mg/kg gemcitabine twice weekly showed no response in all six flank xenografts, confirming that ECM-suppressed cancer cells are resistant to conventional chemotherapeutic agents. In contrast, the ECM-suppressed cells were extirpated in six of eight flank xenografts when treated with DT320 at 45 mg/kg three times weekly (Fig. 1). The difference in response was statistically significant (*p* = 0.0097) using Fisher’s Exact Test.

**Fig. 2:**
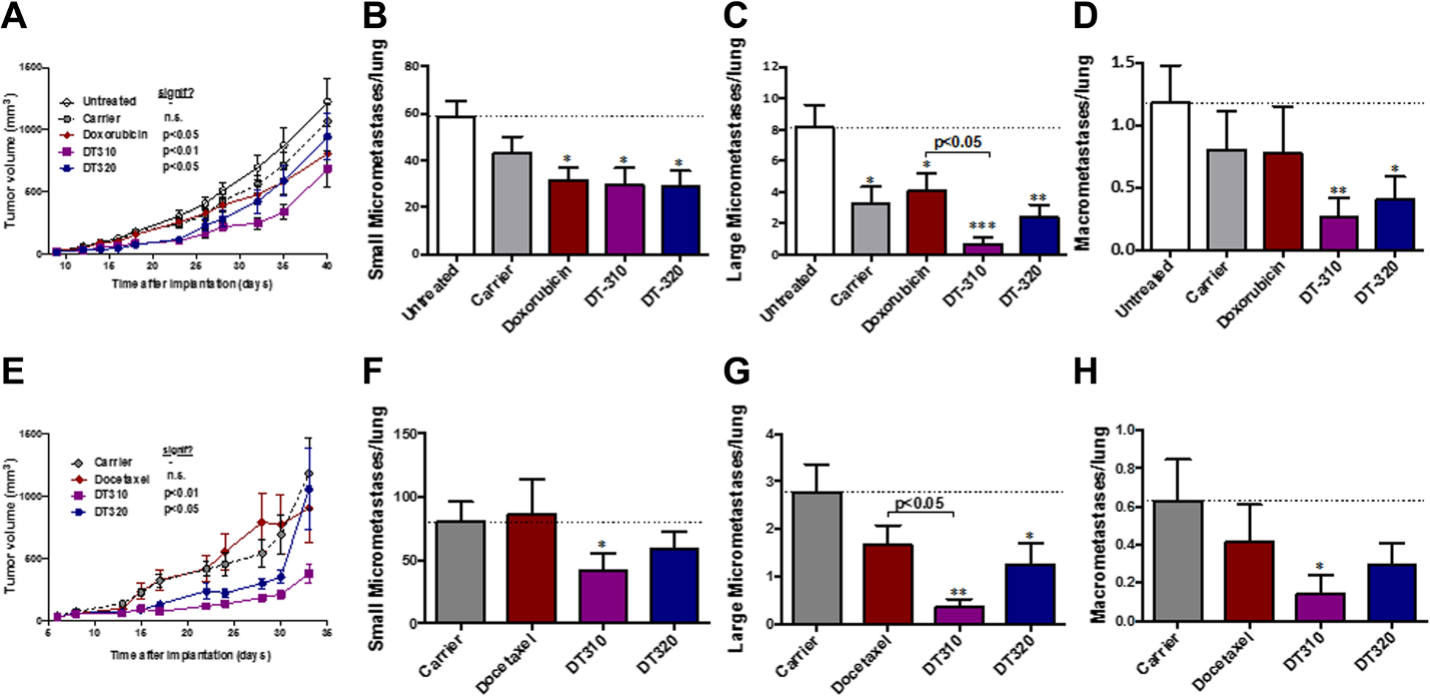
Efficacy of DT310 and DT320 delivery intraperitoneally (**a-d**) or via osmotic pump (**e-h**) in an orthotopic 4T1 syngeneic mouse model of triple-negative breast cancer compared to doxorubicin and docetaxel. An optimized number of 7,500 GFP-labeled cells were injected into the 4th mammary fat pad. Treatment began on day 7 following implantation, which previous studies have shown is when micrometastatic cells begin arriving in the lungs. A and E show the change in volume of the primary tumor in response to treatments. Panels B and F show the effects of treatments on the average number of small lung micrometastases per lung, which are defined as 1–10 cells without vascularization. Panels C and G show the effects of treatment on the average number of large micrometastases per lung, which are defined as clumps of >10 cells without vascularization. Panels D and H show the effects of treatment on the average numbers of lung macrometastases per lung, which are defined as vascularized clumps of cells. Data are means +/−SEM, * = *P <*0.05, ** = *P <*0.01 by two-way ANOVA and repeated measures post-test (panels A and E) or by one-way ANOVA with Tukey post-test (panels B-D; F-H)

Using a more physiologic model in which metastasis occurred naturally would provide a final link in the chain of evidence for our hypothesis that micrometastatic cells can be targeted by taking advantage of their suppressed phenotype. Under such conditions, an effect on both the primary tumor and metastasis should be observed, because small tumors will have a significant fraction of their cells in contact with the normal ECM, at least in an implant model. A modified version of the 4T1 triple negative breast cancer allograft model, which naturally forms micrometastases in the lung [39], was used to test our drugs. It was found that DT310 and DT320 given systemically either by intraperitoneal (i.p.) injection, or by osmotic pump resulted in significant reduction in primary tumor growth vs. untreated animals or animals treated with a maximal tolerated dose of an agent of choice (Fig. 2a and e). Doxorubicin had a small effect when administered i.p., but docetaxel, which is generally effective against triple–negative human breast cancer, had little effect on the 4T1 primary tumors when administered i.p. (Fig. 2a and e). The number of single micrometastatic cells and small micrometastases was reduced about 40 % by all agents with i.p. administration (Fig. 2b) but only DT310 had a significant effect with osmotic pump administration (Fig. 2f). However, the number of large multicellular micrometastases and vascularized macrometastases to the lung were also reduced by all DT agents, but most sharply by DT310 (Fig. 2C, D, G and H), whereas doxorubicin and docetaxel had only minimal effects. Thus, our agents possess *in vivo* anti-metastatic activity, as designed, and were considerably more effective than two standard chemotherapeutic agents used to treat triple–negative breast cancer. None of our agents resulted in weight loss in tumor-bearing animals, indicating low systemic toxicity (data not shown).

### Identification of analogs and potential targets

Analogs of the lead compounds were identified by two methods–simple visual examination of structures and selection of similar appearing structures, and an algorithmic approach based on analysis of molecular shape in three dimensions. A number of structurally similar compounds were arbitrarily selected for activity studies, mostly based on price and commercial availability. The algorithmic method was more efficient than the visual method; 11 out of 15 suggestions by the algorithmic method proved to have activity, whereas only 15 of 43 selected by visual examination proved to be active (*p* = 0.0154; Fisher’s exact test). The results of these studies are shown in Additional file 1: Table S1. Interestingly, none of the “hits” proved to be as active as the original two compounds identified by screening. Table 4 summarizes the potential targets identified by the algorithmic approach as being capable of interacting with the drugs. Considerable overlap was noted between the analog and mechanism/target lists for DT310 and DT320, which was expected because of the similarities in structures. First, suramin, a larger sulfonic acid analog of DT310/DT320, is a potent protein tyrosine phosphatase (PTP) inhibitor [45], as are other similar DT310/DT320 analogues. Next, analogues of both DT310 and DT320 have been found to be inhibitors of DNase gamma (IC50 ~ 3 μM), an enzyme involved in apoptotic cell death [46]. Compounds with similar structures as DT310/DT320 have also been found to inhibit matrix metalloproteinases (MMP) and this would be expected to have a strong association with cancer and the extracellular matrix; though many of the most active MMP inhibitors to date have a hydroxamic acid moiety, absent in our compounds [47]. Finally, analogues of both DT310 and DT320 have been found to inhibit carbonic anhydrases (CA), many of which are involved in cancer growth. Although many active CA inhibitors are sulfonamides, DT310 and DT320 are sulfonic acids [48]. This data indicates that there are a number of potential novel mechanistic targets for DT310/DT320, which will be explored in future studies.

**Table 4:** Identification of potential targets from known targets of the 50 compound analogs with the highest similarity scores

Compound	Targets
DT320	Matrix Metalloproteinases (31)^a^
	Adenosine A1 receptor (7)
	Adenosine A2 receptors (12)
	Carbonic Anhydrases (5)
	Protein-tyrosine phosphatase (4)
	Unspecified (4)
	Adenosine A3 receptor (2)
	Dual specificity protein phosphatase 6 (2)
	ADAM17, Aminopeptidase N, ATP-binding cassette sub-family C member 8, Beta-TC6, Glyceraldehyde-3-phosphate dehydrogenase liver, Glycogen synthase kinase-3 beta, Microbial collagenase, PARP1, (all 1)
DT310	Matrix Metalloproteinases (27)
	Adenosine A1 receptor (11)
	Adenosine A2 receptors (12)
	Carbonic Anhydrases (5)
	Protein-tyrosine phosphatase (4)
	Unspecified (4)
	Adenosine A3 receptor (2)
	Dual specificity protein phosphatase 6 (2)
	ADAM17, Aminopeptidase N, ATP-binding cassette
	sub-family C member 8, Beta-TC6, Glyceraldehyde-3-phosphate dehydrogenase liver, Glycogen synthase kinase-3 beta, Microbial collagenase, PARP1, (all 1)

^a.^Numbers in parentheses indicate the number of instances that the specific target came up in CHEMBL searches

### Mechanistic studies

To elucidate the mechanism of DT310 and DT320, cell cycle analysis and Western blotting was performed. The proteins for Western blot were selected from preliminary results using reverse phase protein array (RPPA) for DT320 and DT321 treated MDA-MB-435 and T24 cells. DT321 is a less active analog of DT320 and was used as positive control to confirm the finding of DT320. Of the 204 proteins in the array, 177 were found to be expressed, and two clusters were identified that were differentially expressed between the two DT drugs in both cell lines and the conventional chemotherapeutic agents. Proteins within these clusters showing the largest differences in protein expression and that were related to known anticancer drug action were confirmed by Western blot. Docetaxel was used as a positive control because it is known to induce a cell cycle arrest and apoptosis signature.

**Fig. 3:**
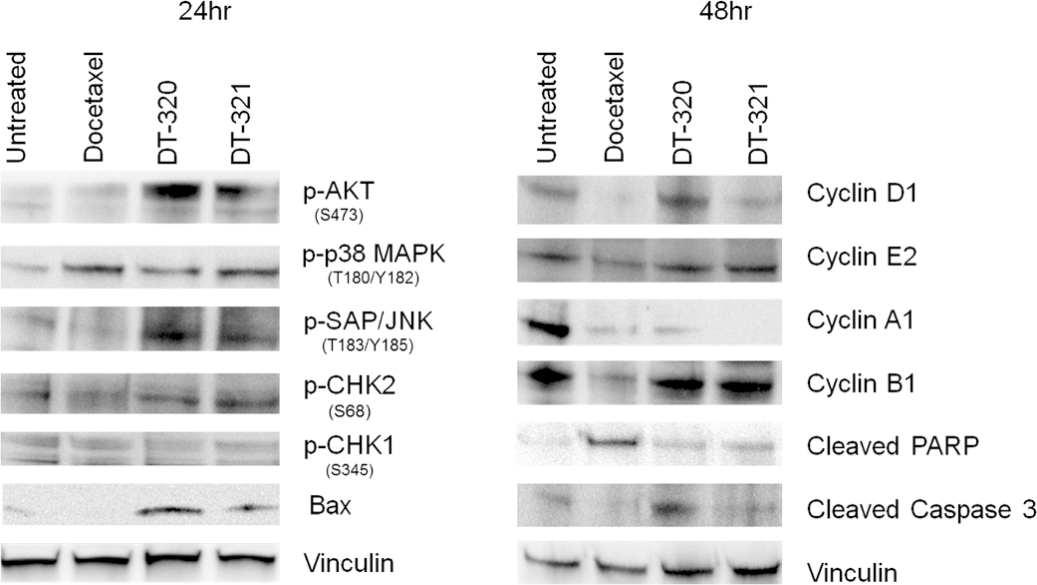
Western blots of protein levels in MDA-MB-435 cells treated with DT agents in comparison to docetaxel 24 and 48 h after treatment. Vinculin was used as a loading control. CHK1 was used as a negative control, since it was not differentially expressed on the RPPA

**Fig. 4:**
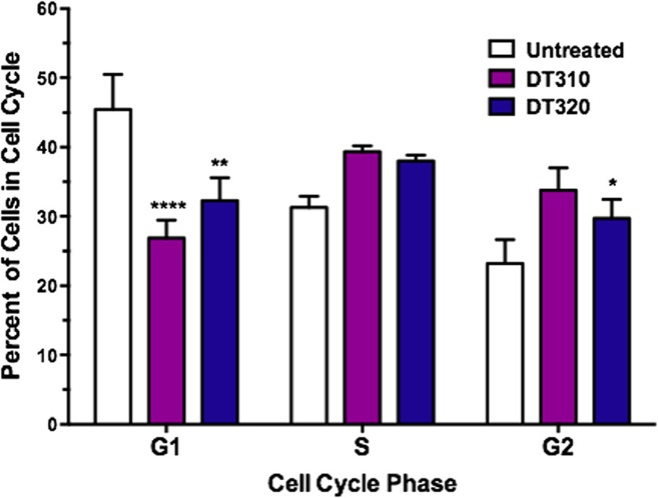
Cell cycle analysis of DT310 and DT320 treatment of MDA-MB-435 cells. Cells were treated 24 h with 100 μM followed by Propidium iodide staining and flow cytometry. Data are means +/−SEM of triplicate samples. * = *P <*0.05, ** = *P <*0.01, **** = *P <*0.001 by one-way ANOVA with Dunnett’s post-test

In general, after 24 h exposure to DT320, changes in the expression of cell signaling proteins were observed; while after 48 h exposure a signature of altered cell cycle proteins and of cell death were observed. Specifically, both of the DT compounds induced a stress response as seen by up-regulation of pAKT at 24 h, whereas docetaxel did not (Fig. 3). Both compounds also up-regulated p-p38 MAPK; pSAP/JNK was also induced by the DT compounds, but not by docetaxel (Fig. 3). pCHK2, which inhibits progression through the S/G_2_ checkpoint in response to DNA damage, was mildly induced by the DT compounds at 24 h, whereas neither of the DT compounds nor docetaxel induced CHK1. The DT compounds also induced the pro-apoptotic protein Bax at 48 h (Fig. 3). DT320 induced Cyclin D1 (CCND1), whereas docetaxel down-regulated this protein. All the compounds, including docetaxel, sharply down-regulate Cyclin A1 (CCNA1), indicating a common action at the S-phase checkpoint. Docetaxel down-regulated cyclin B1 (CCNB1), indicating inhibition of mitosis (Fig. 3). This was not seen with DT compounds. Cell cycle analysis was performed in response to DT310 and DT320 (Fig. 4). DT compounds induced a decrease of the number of cells in G_1_ phase and an increase of cells in S and G_2_ phases. This data suggests that the DT compounds are inducing a mild S/G_2_ cell cycle arrest. Further, the DT drugs had little effect on PARP at 48 h, indicating little activity toward inhibition of DNA repair, unlike docetaxel. An increased signal for cleaved caspase 3 at 48 h reinforces the idea that DT320 also induces apoptosis (Fig. 3). Overall these results suggest that DT compounds induce a stress response unlike docetaxel, and induce a mild cell cycle arrest resulting in a late signature of apoptosis.

### Effect of DT compounds on cancer stem cells

**Fig. 5:**
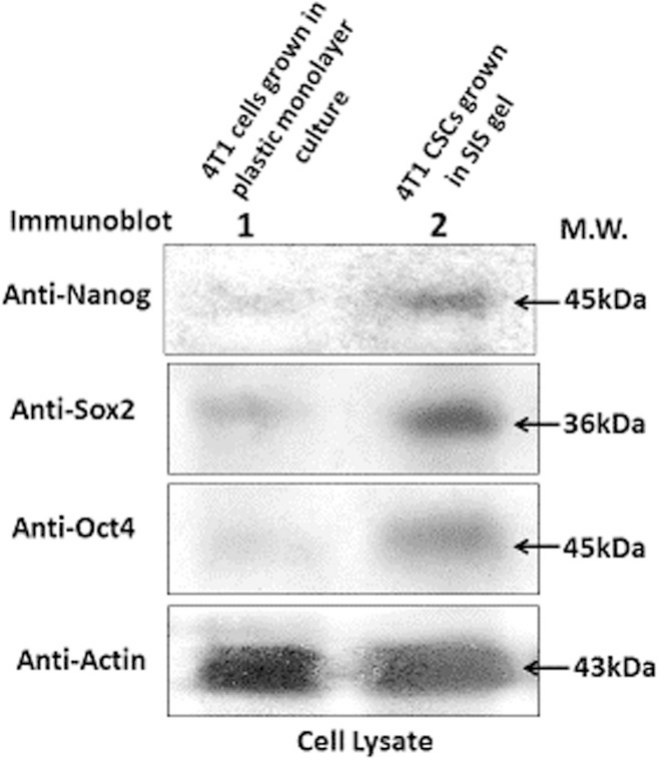
Western blots of pluripotency marker levels in unsorted 4T1 cells grown in plastic monolayer culture (lane 1) and sorted 4T1 CSCs grown in suspension culture and transferred to SISgel (lane 2)

Because cancer stem cells are highly resistant to conventional chemotherapy and are thought to initiate tumors [49], the activity of the DT series of drugs was also checked against cancer stem cells obtained from the 4T1 line. As shown in Table 5, 4T1 stem cells in suspension culture were shown to be highly resistant to doxorubicin, yet surprisingly sensitive to DT310 and DT320 (Table 5). As expected, Orange #1, an inactive analog of DT310 and DT320, showed little activity toward 4T1 stem cells (EC_50_ = 398 μM). For comparison the EC_50_ values of DT310, DT320, and doxorubicin were determined in 4T1 parental cells plated on plastic. Because this assay targeting stem cells was performed in suspension culture, we also tested whether SISgel differentiated the stem cells. As indicated by the presence of pluripotency markers (Nanog, Sox2, Oct4), the cells retained their pluripotency markers on SISgel (Fig. 5). When grown on SISgel, the EC_50_ for DT320 was 71 μM, vs 50 μM in suspension culture, indicating that the SISgel had little effect on the sensitivity of the 4T1 cancer stem cells toward the DT agents. The 4T1 stem cells could not be tested on Matrigel or on plastic because they differentiate and are therefore no longer stem cells. 4T1 cancer stem cells were much more sensitive to the DT agents, particularly to DT320, than to conventional chemotherapeutic drugs.

**Table 5:** EC_50_ and fold change for DT310, DT320, and doxorubicin on parental 4T1 or 4T1 stem cells

Treatment	Parental EC_50_ 4T1 cell line grown on plastic surface	EC_50_ 4T1 stem cells in suspension culture	Fold change
DT310	14.4 μM	141 μM	9.8
DT320	19.7 μM	50 μM	2.5
doxorubicin	4.7 μM	630 μM	134.0

For control, the EC_50_ of an inactive analog of DT310 and DT320, Orange #1, was also determined (EC_50_ = 398 μM) on 4T1 stem cells

## Discussion

In this communication we describe a set of compounds that were identified on the basis of a novel screening assay with the intent of targeting dormant micrometastatic cells. These “ticking time bombs” can often be identified in the tissues of cancer patients [12], where they are assumed to be the source of delayed metastasis [13–18]. A major reason for the suppression or dormancy of micrometastatic cells is that they are unable to overcome the suppressive effects of the normal ECM [11]. To address this, our screen compared the activities of the diversity compound library with cells grown on SISgel, which is prepared from normal ECM, and cells grown on a plastic surface, where the cells avoid the effect of normal ECM. Our logic was that because cancer cells that escape from circulation can end up in any organ and be suppressed, the normal ECM preparation does not need to be organ-specific. We therefore used SISgel, which we earlier showed to suppress the malignant phenotype of cancer cells from different tissues [32]. For identification of potential hits, we needed to compare the sensitivity of suppressed cells to cells that were not suppressed, thus comparison of activities of compounds in cells grown on SISgel and a plastic surface should identify agents that could potentially target micrometastatic cancer cells suppressed by normal ECM. Growing cells on “cancer friendly” ECM, Matrigel, was not required for comparison, because drug sensitivities of cancer cells grown on a plastic surface and Matrigel were either similar, or the cells were more resistant on Matrigel [30]. Out of around 3,000 compounds in the “NCI diversity set”, we identified two leads we call DT310 and DT320. The leads were not highly cytotoxic; therefore, we also screened a second commercially available library of 10,000 drug-like compounds, with the goal to identify more cytotoxic leads. Although we discovered several additional “hits” such as DT340 and DT350, they all had dose-limiting toxicity in mice. This was not the case for DT310 and DT320, which had very low toxicity.

DT310 and DT320 were tested in an orthotopic flank model in which the cells were co-injected with SISgel. We had earlier shown that the suppressed phenotype is established within 24 h [30] and that cells co-injected with SISgel as flank xenografts can maintain a suppressed or dormant state for several weeks before sometimes emerging as active tumors even though the SISgel itself rapidly disappears [33]. We showed that the lead compounds are active *in vivo,* in the flank xenograft model, as well as in the cell culture model (Fig. 1), thus supporting the reasoning behind the screening. However, this model is still somewhat artificial in that an exogenous agent (SISgel) is used to suppress the malignant phenotype or induce dormancy. We therefore tested these compounds in a physiological orthotopic model of metastasis, in which the normal extracellular matrix is provided by the mouse lung. In this model, the lead compounds prevented growth of micrometastatic cells into macroscopic tumors. In addition, they outperformed both doxorubicin and gemcitabine in inhibiting growth of the primary tumor. This apparently paradoxical result supports the hypothesis that normal extracellular matrix inhibits tumor growth, and that these agents act at the interface where tumor cells are responding to the inhibitory effects of normal ECM. Whether this effect would be noted with spontaneous tumors is unclear because a recent paper showed that implanted cells show different pharmacokinetics than do spontaneous tumors, most likely because of differences in the microenvironment and ECM [50].

Even though the EC_50_ is higher against the cancer stem cell-enriched population than against the parental cell line, the differential activity toward the 4T1 stem cell-containing population *in vitro* suggests that these compounds could be effective against the rare cancer stem cells that make their way to remote tissues. More research will be necessary to establish whether these compounds actually target cancer stem cells *in vivo*. The relatively high EC_50_ does not necessarily indicate a lack of activity because many effective anticancer agents are only active in the millimolar range [51], and the low toxicity of these compounds should enable delivery of high enough concentrations to be effective.

The target and mode of action are not entirely clear, but the DT compounds appear to induce replicative inhibition (dormancy) and apoptosis in cancer cells interacting with normal extracellular matrix. This is suggested by the action *in vitro*, the effect on primary tumor growth, the efficacy against SISgel flank xenografts, and is confirmed by the induction of cell cycle arrest and late up-regulation of cleaved caspase-3 (Fig. 3). Their mode of action appears to be different from docetaxel and other chemotherapeutics. While the DT drugs do not seem to affect DNA repair, they do seem to affect the cell cycle. One intriguing possibility is suggested by the upregulation of p38 MAPK, which has been implicated in producing dormancy. Also, whether the mode of action is exactly the same in natural metastasis as in the flank model is not clear. A number of potential targets are suggested from the computational similarity studies. Further research will be required to identify whether any single or combination of mechanisms is involved in the mode of action and to identify the target protein or proteins.

These drugs represent a novel approach to targeting metastasis at a particularly vulnerable stage of single micrometastatic cells. Using this approach, tumor heterogeneity, a key factor in chemotherapeutic resistance, can be minimized and the tumor cells eradicated before they reactivate to form macrometastases or recurrent tumors. Although the potency of these leads is above the nanomolar range the current the current paradigm of drug development suggests is desirable, many effective compounds are active in the micromolar range and uncritical application of the “nanomolar rule” could reject highly effective compounds [51]. It is not clear the degree to which the *in vitro* assay of potency actually reflects the *in vivo* activity, and the efficacy and low toxicity shown in the orthotopic model suggests these compounds could prove useful clinically.

Recent studies suggest that potentially metastatic cells enter circulation early in tumor growth and disseminate to the body tissues, where most die within a relatively short time [13]. A few can remain dormant for long periods of time, up to decades, and a very few activate and form a metastatic tumor that then begins disseminating a second generation of metastatic cells [52–54]. Delayed metastasis, or even surgery-induced activation of dormant cancer cells, could potentially be eliminated by targeting dormant micrometastatic cells [54]. These drugs also appear to be sufficiently non-toxic that a course of administration to most or all cancer patients undergoing therapy would be possible, if they prove to be effective in humans.

## Conclusions

(1) Cancer cells on a normal ECM have a different sensitivity to drugs than do cancer cells on plastic in conventional tissue culture. (2) Drugs that are more potent against cells on normal ECM appear to target dormant micrometastatic cells. This is shown in both a novel flank xenograft using exogenous normal ECM as well as in a physiologic model of metastasis in which no exogenous ECM was used. The findings suggest that these drugs could be useful in targeting micrometastatic cells present in patient tissues at the time of diagnosis, thereby preventing recurrence at a later date.

## Additional file

Additional file 1: Table S1. Analogs of compounds identified from screen using visual and algorithmic techniques.

## Abbreviations

CSC: Cancer stem cell
EC_50_: Effective concentration at which cell kill was 50 %
ECM: Extracellular matrix
MTD: Maximum tolerated dose
SIS: Small intestine submucosa
SISgel: A gel-forming normal matrix material prepared from SIS
