# Supplementary material for: Identification of novel drugs to target dormant micrometastases
Source: BMC Cancer. 2015 May 14;15:404. doi: 10.1186/s12885-015-1409-4 (PMC4434572; doi:10.1186/s12885-015-1409-4)
Supplement: Additional file 1: Table S1. — Analogs of compounds identified from screen using visual and algorithmic techniques. [file 12885_2015_1409_MOESM1_ESM.docx]

Table S1. Analogs of compounds identified from screen using visual and algorithmic techniques.

| Compound Common Name | CAS No. | Cpd. Family | ID Method | IC_50_-p | IC_50_-S | SR | Hit |
| --- | --- | --- | --- | --- | --- | --- | --- |
| 2-2-dihydroxy-1-1-azo-naphthalene-3-3-6 | 76877-41-3 | DT310/320 | Visual | 213 | 111 | 1.9 | NO |
| 8-(p-Sulfophenyl)theophylline hydrate | 80206-91-3 | DT310/320 | Visual | 346 | 140 | 2.5 | NO |
| Acid Blue 1 | 116-95-0 | DT310/320 | Visual | 318 | 194 | 1.6 | NO |
| Acid Blue 29 | 5850-35-1 | DT310/320 | Algorithm | 212 | 122 | 1.7 | NO |
| Acid Dye (Azo Red) | 5858-64-0 | DT310/320 | Visual | 23 | 21 | 1 | NO |
| Acid Green 3 | 4680-78-8 | DT310/320 | Visual | 492 | 666 | 0.74 | NO |
| Acid Orange 7 | 633-96-5 | DT310/320 | Visual | 114 | 400 | 0.29 | NO |
| Acid Red 1 | 3734-67-6 | DT310/320 | Algorithm | 33 | 13 | 2.6 | YES |
| Acid Red 26 | 3761-53-3 | DT310/320 | Visual | 154 | 604 | 0.26 | NO |
| Acid Red 37 | 302912-22-7 | DT310/320 | Algorithm | 85 | 51 | 1.7 | YES |
| Acid Red 60 Chromotrope 2B | 5850-64-6 | DT310/320 | Visual | 26 | 22 | 1.2 | NO |
| Acid Violet 49 | 1694-09-3 | DT310/320 | Visual | 64 | 86 | 0.75 | NO |
| Alizarin Green | 4857-81-2 | DT310/320 | Visual | 414 | 621 | 0.67 | NO |
| Amaranthe | 915-67-3 | DT310/320 | Visual | 33 | 26 | 1.3 | NO |
| Arsenazo III | 1668-00-4 | DT310/320 | Algorithm | 47 | 34 | 1.4 | NO |
| Beryllon II | 51550-25-5 | DT310/320 | Visual | 94 | 33 | 3 | YES |
| Brilliant Crocein Moo | 5413-75-2 | DT310/320 | Algorithm | 82 | 47 | 1.8 | YES |
| Calichrome | 3810-39-7 | DT310/320 | Visual | 464 | 375 | 1.2 | NO |
| Chromotrope 2B | 548-80-1 | DT310/320 | Visual | 71 | 40 | 1.8 | YES |
| Chromotrope 2R | 4197-07-3 | DT310/320 | Visual | 92 | 94 | 0.98 | NO |
| Chromotrope 6B | 4197-09-5 | DT310/320 | Visual | 71 | 34 | 2.1 | YES |
| Chromotrope F4B | 3746-79-0 | DT310/320 | Visual | 90 | 50 | 1.8 | YES |
| Comassie Brilliant Blue | 6104-58-1 | DT310/320 | Visual | 201 | 125 | 1.6 | NO |
| DT-310 | 5858-33-3 | DT310/320 | Screen | 87 | 36 | 2.4 | YES |
| DT-320 | 5850-63-5 | DT310/320 | Screen | 78 | 9 | 9.0 | YES |
| Hydroxy Naphthol Blue | 165660-27-5 | DT310/320 | Visual | 146 | 72 | 2.0 | YES |
| HydroxyNapthol Blue | 63451-35-4 | DT310/320 | Visual | 135 | 64 | 2.1 | YES |
| Light Green SF Yellowish | 5141-20-8 | DT310/320 | Visual | 631 | 315 | 2 | NO |
| Mordant Blue 9 | 3624-68-8 | DT310/320 | Algorithm | 323 | 176 | 1.8 | NO |
| New Coccine | 2611-82-7 | DT310/320 | Algorithm | 43 | 27 | 1.6 | YES |
| nitrazine Yellow | 5423-07-4 | DT310/320 | Algorithm | 65 | 33 | 2.0 | YES |
| Orange 1 | 523-44-4 | DT310/320 | Visual | 657 | 1,199 | 0.55 | NO |
| Palatine Chrome Green | 3564-28-1 | DT310/320 | Visual | 74 | 70 | 1.1 | NO |
| Ponceau 3R | 3564-09-8 | DT310/320 | Visual | 146 | 111 | 1.3 | NO |
| Ponceau 6R | 5850-44-2 | DT310/320 | Visual | 124 | 107 | 1.2 | NO |
| Ponceau SX | 4548-53-2 | DT310/320 | Visual | 208 | 54.1 | 3.8 | YES |
| Ponceau Xylidine | 3761-53-3 | DT310/320 | Algorithm | 38 | 15 | 2.6 | YES |
| Pontacyl Carmine 2B | 6625-46-3 | DT310/320 | Visual | 53 | 24 | 2.2 | YES |
| PPADS | 149017-66-3 | DT310/320 | Visual | 419 | 293 | 1.4 | NO |
| Procion Red Mx - 5B | 17804-49-8 | DT310/320 | Algorithm | 77 | 57 | 1.4 | NO |
| Remazol Brilliant Violet 5R | 12226-38-9 | DT310/320 | Algorithm | 172 | 93 | 1.9 | NO |
| Sulfanazo II | 68504-35-8 | DT310/320 | Visual | 57 | 34 | 2 | YES |
| Sulfanilic Acid Azochromotrop | 23647-14-5 | DT310/320 | Algorithm | 38 | 19 | 2.0 | YES |
| Sulfochlorophenol S | 108321-09-1 | DT310/320 | Algorithm | 46 | 23 | 2.0 | YES |
| Sunset Yellow FCF | 2783-94-0 | DT310/320 | Algorithm | 67 | 31 | 2.2 | YES |
| Suramin | 129-46-4 | DT310/320 | Visual | 321 | 226 | 1.4 | NO |
| Trypan Blue | 72-57-1 | DT310/320 | Algorithm | 159 | 36 | 4.5 | YES |
| Xylidyl Blue 1 | 14936-97-1 | DT310/320 | Algorithm | 106 | 45 | 2.4 | YES |
